# Supplementary material for: Comparative genomics provides new insights into the diversity, physiology, and sexuality of the only industrially exploited tremellomycete: Phaffia rhodozyma
Source: BMC Genomics. 2016 Nov 9;17:901. doi: 10.1186/s12864-016-3244-7 (PMC5103461; doi:10.1186/s12864-016-3244-7)
Supplement: Additional file 6: — List of orphan genes with links to PFAM (related to Additional file 1: Table S1). (ZIP 1428 kb) [file 12864_2016_3244_MOESM6_ESM.zip › PFAM_UNIQUE/G01838_P.html]

#                                                                            --- full sequence --- -------------- this domain -------------   hmm coord   ali coord   env coord
  
# target name        accession   tlen query name           accession   qlen   E-value  score  bias   #  of  c-Evalue  i-Evalue  score  bias  from    to  from    to  from    to  acc description of target
  
#------------------- ---------- ----- -------------------- ---------- ----- --------- ------ ----- --- --- --------- --------- ------ ----- ----- ----- ----- ----- ----- ----- ---- ---------------------
  
G01838\_P             -            837 CKAP2\_C              PF15297.1    353       9.6    3.6   7.7   1   1     0.024        29    2.0   7.7   206   255   527   576   505   636 0.58 - Cytoskeleton-associated protein 2 C-terminus  
G01838\_P             -            837 DUF885               PF05960.6    549       8.4    4.1   8.3   1   1     0.011        16    3.1   8.3    15   142   536   664   518   677 0.71 - Bacterial protein of unknown function (DUF885)  
G01838\_P             -            837 Med19                PF10278.4    178       4.4    5.6   7.7   1   1     0.031        17    3.7   7.7   116   170   523   582   495   592 0.37 - Mediator of RNA pol II transcription subunit 19  
G01838\_P             -            837 Merozoite\_SPAM       PF07133.6    173       2.8    6.5  12.4   1   1     0.021         7    5.2  12.4    29    94   519   583   517   601 0.67 - Merozoite surface protein (SPAM)  
G01838\_P             -            837 WW                   PF00397.21    31     2e-09   35.8   0.3   1   1   8.3e-12     5e-09   34.6   0.3     3    31   225   254   223   254 0.89 - WW domain
